# Safety and Immunogenicity of Inactivated and Recombinant Protein SARS-CoV-2 Vaccines in Patients With Thyroid Cancer

**DOI:** 10.3389/fimmu.2022.855311

**Published:** 2022-05-13

**Authors:** Yuling Han, Jiaxin Yang, Danshuang He, Yang Feng, Xiaoman Liu, Yu Min, Shenghao Fan, Guobing Yin, Daixing Hu

**Affiliations:** Department of Breast and Thyroid Surgery, The Second Affiliated Hospital of Chongqing Medical University, Chongqing, China

**Keywords:** Coronavirus disease 2019 (COVID-19), severe acute respiratory syndrome coronavirus 2 (SARS-CoV-2), inactivated SARS-CoV-2 vaccine, immunogenicity, safety

## Abstract

**Background:**

This study aimed at assessing the safety and immunogenicity of SARS-CoV-2 vaccines in patients with thyroid cancer.

**Methods:**

This observational study included thyroid cancer patients between April 1, 2021, and November 31, 2021, in the Second Affiliated Hospital of Chongqing Medical University. All participants received at least one dose of the SARS-CoV-2 vaccine. SARS-CoV-2 IgG was tested, and the interval time between the last dose and humoral response test ranged from <1 to 8 months. The complications after SARS-CoV-2 vaccines were recorded.

**Results:**

A total of 115 participants at least received one dose of SARS-CoV-2 vaccines with a 67.0% IgG-positive rate. Among them, 98 cases had completed vaccination, and the positivity of SARS-CoV-2 IgG antibodies was 96% (24/25) with three doses of ZF2001. SARS-CoV-2 IgG antibodies’ positivity was 63.0% (46/73) of two doses of CoronaVac or BBIBP-CorV vaccine. Additionally, after 4 months of the last-dose vaccination, the IgG-positive rate (31.6%, 6/19) significantly decreased in thyroid cancer patients. The IgG-positive rate (81.0%, 64/79) was satisfactory within 3 months of the last-dose vaccination. Ten (10.2%) patients had side effects after SARS-CoV-2 vaccination. Among them, two (2.0%) patients had a fever, five (5.1%) patients had injection site pain, one (1.0%) patient felt dizzy, and one patient felt dizzy and had injection site pain at the same time.

**Conclusion:**

SARS-CoV-2 vaccines (CoronaVac, BBIBP-CorV, and ZF2001) are safe in thyroid cancer patients. The regression time of SARS-CoV-2 IgG is significantly shorter in thyroid cancer patients than in healthy adults. Therefore, a booster vaccination dose may be earlier than the systematic strategy for thyroid cancer patients.

## Introduction

Coronavirus disease 2019 (COVID-19) is caused by severe acute respiratory syndrome coronavirus 2 (SARS-CoV-2) (1). Since the global COVID-19 pandemic, more than 306 million individuals worldwide have been infected, while 5.5 million COVID-19-related deaths were recorded worldwide (January 9, 2022). Currently, no specific medicine is available for the treatment of SARS-CoV-2. Thus, vaccination is essential to preventing COVID-19 (2–4).

There are multiple types of vaccines in research and development. The mRNA vaccine was widely used in European and American countries (5, 6), and inactivated vaccines and recombinant protein vaccines have been used more often in China (7, 8). mRNA vaccine BNT162b2 (Pfizer BioNTech; Mainz, Germany) successfully prevented COVID-19 with more than 90% efficacy (9). Several studies demonstrated that inactivated vaccines had good immunogenicity of SARS-CoV-2 with a low complication rate (10–12). However, most people enrolled in these studies are healthy adults, while patients with immune-compromised or cancers were excluded. Taghizadeh-Hesary et al. found that COVID-19 patients with cancer history had a higher rate of mechanical ventilation and mortality than non-cancer history patients (13). A previous study reported that routine COVID-19 testing may be necessary for cancer patients with comorbidities or older age but may not benefit for most cancer patients (14). A multicenter, prospective observational study in Iran analyzed the seroprevalence of the SARS-CoV-2 IgG antibody in a cancer population referred for vaccination, which indicated that almost 20% of cancer patients may suffer from an asymptomatic disease only in serological assessment, and seropositivity was significantly greater in women consistent with higher rates in breast cancer and gynecologic cancers patients. Not only that, gender and prior history of COVID-19 were independently associated with seropositivity prior to vaccination (15). Soroosh et al. elaborated on the concerns and issues of oncology centers during COVID-19 outbreaks, including limited resources of healthcare systems and lack of appropriate guidelines (16). Thus, some scholars called for more data on COVID-19 vaccinations in cancer patients, such as the vaccine efficacy data, the durability of vaccine protection compared to the general population, and the immune response among cancer patients (17). Recent research investigated the relationship between cancer patients and the BNT162b2 vaccine, indicating that more than 90% of cancer patients exhibited seropositivity. Meanwhile, the antibody titers of cancer patients were significantly lower than those of healthy controls (18). Shmueli et al. found that the seropositivity rate among patients with cancer and healthy controls was 84.1% and 98.9% after the second dose of BNT162b2 vaccination, respectively (19). These studies included multiple cancer types, such as gastrointestinal, lung, breast, genitourinary, and melanoma. However, no relevant research has been reported on the safety and immunogenicity of SARS-CoV-2 vaccines in patients with thyroid cancer.

Thyroid cancer is the 11th among all malignant tumors with rapid growth of incidence (20). As of 2020, the number of new cases of thyroid cancer was more than 580,000 around the world. Presumably, the incidence of thyroid cancer may rank fourth among all kinds of malignant tumors in 2030 (21). Therefore, we need to explore the related issues of SARS-CoV-2 vaccines in thyroid cancer patients. This study assessed the safety and immunogenicity of SARS-CoV-2 vaccines in this particular population.

## Materials and Methods

### Study Design

We did an observational study of pathological diagnosis with thyroid cancer patients between April 1, 2021, and November 31, 2021, in the Department of Breast and Thyroid Surgery, The Second Affiliated Hospital of Chongqing Medical University. In this study, 115 thyroid cancer patients received at least one dose of the SARS-CoV-2 vaccine, and 98 cases had complete vaccination. Most individuals in China received inactivated vaccines (CoronaVac and BBIBP-CorV) and recombinant protein vaccines (ZF2001). Thus, this study analyzed the three types of vaccines above. Basic clinical characteristics of age, gender, and comorbidities (diabetes, hypertension, hyperlipidemia, and Hashimoto’s thyroiditis) were obtained from patients’ medical records. The study protocol was approved by the local institutional ethics board of the Second Affiliated Hospital of Chongqing Medical University.

### Serology Assays

All patients detected the SARS-CoV-2 antibody IgG by chemiluminescence (Bioscience Diagnostic Technology Co., Ltd, approved by the China National Medical Products Administration; approval number 20203400183 for IgG) according to the manufacturer’s instructions. Peripheral blood samples were tested on the machine after centrifugation. Values more than 1.0 of the cutoff index were indicated as SARS-CoV-2 antibody IgG positive.

### Safety

After patients’ admission, side effects (injection-site symptoms and systemic symptoms) of SARS-CoV-2 vaccines were obtained by clinical history-taking. We asked patients using specific yes/no questions regarding local reactions (pain at the injection site, redness, and swelling) and systemic symptoms (fever >37.3°C, dizzy, fatigue, headache, chills, myalgia, and nausea).

### Local Epidemic Overview

Chongqing is a provincial administrative unit, which is located in southwest China with more than 30 million population. The COVID-19 cumulative confirmed cases were 691 with 6 deaths (April 4, 2022). Thus, the prevention and control policy for COVID-19 were effective. A total of 19 COVID-19 cases were found during the period in this study (from April 1, 2021, to November 31, 2021), including eight local confirmed cases and 11 imported cases. None of the thyroid cancer patients in this study were infected with COVID-19.

### Statistical Analysis

Age is displayed as median ± standard deviation and analyzed by unpaired t-test. The analysis of categorical variables used Pearson χ² test or Fisher’s exact test. SPSS 26 (IBM Corp., Armonk, NY, USA) and GraphPad Prism version 7.0 were used for statistical analysis. p values less than 0.05 were assigned significance.

## Results

### Participants’ Characteristics

From April 1, 2021, to November 31, 2021, a total of 115 participants at least received one dose of inactivated vaccine and recombinant protein vaccine with a 67.0% IgG-positive rate. Among them, the positivity of SARS-CoV-2 IgG antibodies was 96.0% (24/25) in patients with three doses of ZF2001. In contrast, 75.0% (6/8) and 0% (0/4) patients had positive antibodies who received two doses and one dose of ZF2001, respectively. This study included two inactivated vaccines; patients who received CoronaVac or BBIBP-CorV vaccine two doses and one dose had 63.0% (46/73) and 20.0% (1/5) positivity of SARS-CoV-2 IgG, respectively. Subsequently, we analyzed the immunogenicity and safety of completed vaccination patients (98 cases). Among the 98 thyroid cancer patients, one 45-year-old woman was also diagnosed with breast cancer. Because the chemotherapy is not over, she has not performed surgery now. Fine-needle aspiration was performed on this patient to diagnose thyroid cancer by cytology with BRAF-mutated. The clinicopathological characteristics of the other 97 thyroid cancer patients were as shown in **Table 1**, which showed that the mean age at diagnosis was 40.4 years (range from 20 to 66 years). The ratio of women to men is 2.88:1, with 25 men and 72 women. Forty-two patients underwent total thyroidectomy, and 23 patients received iodine-131 therapy.

**Table 1:** Thyroid cancer patient demographics and clinical characteristics (n = 97).

Characteristics	Results
Age at diagnosis (mean ± SEM, years)	40.4 ± 1.2
≤55 years	84
>55 years	13
Sex (male/female)	25/72
Unilateral thyroid cancer (yes/no)	83/14
Pathological type (papillary/medullary)	95/2
TNM stage (I/II/III/IV)	95/2/0/0
Iodine-131 therapy (yes/no)	23/74
Extent of surgery (total thyroidectomy/lobectomy)	42/55

### Vaccine Safety

Ten (10.2%) patients had at least one side effect after either dose of SARS-CoV-2 vaccination. Among them, two (2.0%) patients had a fever, five (5.1%) patients had pain at the injection site, one (1.0%) patient felt dizzy, and one patient felt dizzy and had pain at the injection site at the same time.

### SARS-CoV-2 Vaccination Immunogenicity

The vaccine’s effectiveness is the most concerning result, which may change current epidemic prevention strategies. Firstly, we analyzed the clinical characteristic between the SARS-CoV-2 IgG-positive group and the negative group (**Table 2**). However, age (p = 0.636), gender (p = 0.341), Hashimoto’s thyroiditis (p = 0.330), hypertension and hyperlipidemia (p = 0.500), and diabetes (p = 0.561) were not statistically significantly different with SARS-CoV-2 IgG generation. Compared with the antibody-positive group, the antibody-negative group had a higher proportion of men (32.1% vs. 22.9%) and a lower proportion of Hashimoto’s thyroiditis (7.1% vs. 14.3%). Then, because of the characterization of our observational study, we found the subsidence condition of SARS-CoV-2 IgG. After 4 months of the last-dose vaccination, the SARS-CoV-2 IgG-positive rate (31.6%, 6/19) significantly decreased in thyroid cancer patients. The opposite is that the SARS-CoV-2 IgG-positive rate (81.0%, 64/79) was satisfactory within 3 months of the last-dose vaccination (**Table 3**). Additionally, none of the thyroid cancer patients in this study were infected with COVID-19. Finally, the clinical characteristics of 28 thyroid cancer patients who were seronegative are presented in **Table 4**.

**Table 2:** The clinical characteristic between the SARS-CoV-2 IgG positive group and the negative group.

	Antibody positive (n = 70)	Antibody negative (n = 28)	P value
Age (mean ± SEM, years)	40.07 ± 1.37	41.29 ± 2.13	0.636
Gender (male/female)	16 (22.9%)/54 (78.1%)	9 (32.1%)/19 (67.9%)	0.341
Hashimoto’s thyroiditis (yes/no)	10 (14.3%)/60 (85.7%)	2 (7.1%)/26 (92.9%)	0.330
Hypertension and hyperlipidemia (yes/no)	11 (15.7%)/59 (84.3%)	6 (21.4%)/22 (78.6%)	0.500
Diabetes (yes/no)	3 (4.3%)/67 (95.7%)	2 (7.1%)/26 (92.9%)	0.561

**Table 3:** The positive rate of the SARS-CoV-2 antibody according to different interval times.

Interval time, months	Antibody positive (N = 70)	Antibody negative (N = 28)	Antibody positive rate (%)
<1	17	6	73.9
1	20	1	95.2
2	19	5	79.2
3	8	3	72.7
4	4	4	50.0
>5	2	9	18.2

**Table 4:** Characteristics of patients who were SARS-CoV-2 antibody negative in thyroid cancer.

Patient no.	Gender	Age, years	Interval time, months	Vaccine company	Comorbidities	Side effect
1	Female	35	<1	ZF2001	–	–
2	Female	37	<1	BBIBP-CorV	–	–
3	Male	25	<1	BBIBP-CorV	Hyperlipidemia	–
4	Female	61	<1	CoronaVac	Hypertension and diabetes	–
5	Female	34	<1	BBIBP-CorV	–	–
6	Male	23	<1	CoronaVac	–	–
7	Female	32	1	BBIBP-CorV	–	–
8	Female	50	2	BBIBP-CorV	–	–
9	Female	26	2	CoronaVac	–	–
10	Female	49	2	CoronaVac	Hypertension	–
11	Female	24	2	BBIBP-CorV		–
12	Female	57	2	BBIBP-CorV		–
13	Female	28	3	CoronaVac		–
14	Male	50	3	BBIBP-CorV	Hashimoto’s thyroiditis	–
15	Female	33	3	CoronaVac	–	–
16	Female	55	4	CoronaVac	–	–
17	Female	42	4	CoronaVac	–	–
18	Male	53	4	BBIBP-CorV	–	–
19	Male	48	4	CoronaVac	Hypertension and diabetes	–
20	Female	36	5	BBIBP-CorV	–	Fever
21	Female	44	5	BBIBP-CorV	Hashimoto’s thyroiditis	–
22	Female	56	5	BBIBP-CorV	–	–
23	Female	40	5	CoronaVac	–	Pain at injection site
24	Male	48	5	CoronaVac	–	–
25	Male	45	6	BBIBP-CorV	–	–
26	Female	34	6	CoronaVac	–	–
27	Male	35	6	BBIBP-CorV	Hyperlipidemia	–
28	Male	56	7	CoronaVac	–	–

## Discussion

Previous studies found that the multiple types of SARS-CoV-2 vaccines had satisfactory safety and immunogenicity in healthy adults (22). Our study focused on the effect of inactivated vaccines (CoronaVac and BBIBP-CorV) and recombinant protein vaccines (ZF2001) in thyroid cancer patients. According to the immunogenicity data, recombinant protein vaccine had a high antibody-positive rate with three doses (24/25, 96.0%), which was significantly higher than in two doses of BBIBP-CorV or CoronaVac vaccine (46/73, 63.0%). In the phase 2 trial, Yang et al. suggested that seroconversion rates of neutralizing antibodies 14 days after the third dose of ZF2001 (25 μg) were 96.6% (143 of 148 participants) in healthy adults, which was very consistent with our findings (23). In a phase 2 trial for BBIBP-CorV in healthy adults, 79.2% (19/24) in the 2-μg group, 87.5% (21/24) in the 4-μg group, and 95.8% (23/24) in the 8-μg group seroconverted by day 14, and the seroconversion rates were 100% in all three groups on day 28 (24). The above data showed good immunogenicity of BBIBP-CorV in healthy adults, which had a big gap in the results of our study. Additionally, Ariamanesh et al. analyzed the immunogenicity of the inactivated SARS-CoV-2 vaccine (BBIBP-CorV) in 364 cancer patients (breast cancer 44%, colorectal cancer 14.6%, upper GI cancers 8.8%, hematologic malignancies 6.6%, head and neck cancer 5.2%, prostate cancer 4.9%), which indicated that the overall seroconversion rate was 86.9% 2 months following vaccination (25). In our study, Sinopharm BBIBP-CorV only had a 25% (1/4) antibody-positive rate in thyroid cancer patients after 2 months of the last dose of vaccination. A recent study focused on the efficacy and safety of the BBIBP-CorV vaccine in breast cancer patients (160 cases), which found that injection site pain and fever were 22.3% and 24.3% in patients, respectively. Meanwhile, they reported that more than 85% of breast cancer patients had SARS-CoV-2 antibodies positive (26). The above study had a higher rate of adverse events than our study. This phenomenon may be attributed to the biological features of thyroid cancer, which is more moderate than breast cancer. A systematic review and meta-analysis demonstrated that cancer patients using activated and inactivated vaccines are safe with high efficacy (88% in patients with solid tumors and 70% in patients with hematologic malignancies; this study was accepted in Frontiers in Endocrinology). However, the intrinsic mechanism has not been very clear, and the number of ZF2001 vaccines received was small in this study. Thus, multicenter, large-sample studies are needed to reveal the phenomenon more comprehensively.

Most thyroid cancer patients were diagnosed by routine physical examinations without any symptoms. Benefiting from this special clinical characteristic of thyroid cancer, although our study was observational, we found several important results of antibody disappearing. This is because if they do not have physical examinations, they should not know the thyroid cancer. In turn, they might not have the SARS-CoV-2 IgG detection. For this reason, we obtained the immunogenicity data of three types of vaccines for as long as 8 months after the last dose of vaccination. This study suggested that the positive rate of SARS-CoV-2 IgG had a significant decrease after 4 months of vaccination. Additionally, the SARS-CoV-2 IgG-positive rate was as high as 95.2% when the interval time between the last dose and SARS-CoV-2 IgG detection was 1 month. A recent phase 2 clinical trial focused on the immunogenicity and safety of the third dose of CoronaVac in healthy adults was published online on December 6, 2021, demonstrating that the neutralizing antibody titers declined substantially 6 months after two doses of CoronaVac. A booster dose of CoronaVac 8 months after a second dose effectively recalled specific immune responses to SARS-CoV-2 with low adverse events (7). The above study recommended the third dose of CoronaVac 8 months after the second dose in healthy adults. However, the immune system of thyroid cancer patients is probably different from that of healthy adults. Whether the timing of a booster dose less than 8 months might call for considerable attention in thyroid cancer patients remains to be studied. We speculate that it may have a better interval between the last and booster doses.

This study analyzed the clinical characteristics between the antibody-positive and antibody-negative groups, although age, gender, Hashimoto’s thyroiditis, hypertension and hyperlipidemia, and diabetes had no statistically significant difference between these two groups. However, the antibody-negative group had a higher proportion of men (32.1% vs. 22.9%) and a lower proportion of Hashimoto’s thyroiditis (7.1% vs. 14.3%) compared with the antibody-positive group. Previous studies found that the male gender was an independent risk factor for negative serological response to multiple SARS-CoV-2 vaccines in the general population with unknown mechanisms (27, 28). We suggested that the proportion difference of gender and Hashimoto’s thyroiditis may have a particular clinical value, which deserves further attention.

Previous studies reported that the adverse events were relatively low for ZF2001, BBIBP-CorV, and CoronaVac in clinical trials, which is consistent with the conclusions of our present study (8, 12, 23). Ten (10.2%) patients had an adverse event in our research, while no severe adverse reaction happened. Shmueli et al. found that the adverse event rate was as high as 55% of the BNT162b2 vaccine (Pfizer, New York, USA, and BioNTech, Mainz, Germany) in patients with solid cancer, including gastrointestinal, breast, lung, melanoma (19). Thyroid cancer patients were not included in that study.

There exist several limitations in the present study. First, this was a single-center observation study in southwest China with a relatively small case. Second, most thyroid cancer patients have a favorable prognosis without chemical therapy or radiation therapy, which has less impact on the whole body and immune system. Therefore, the effect of SARS-CoV-2 vaccines in the context of thyroid cancer is not well understood. We should give enough attention to this topic because of the long*-*term coexistence with SARS-CoV-2. In our study, none of the thyroid cancer patients were infected with COVID-19. We suggest a longer-term follow-up to detect clinical COVID-19 infection rates and outcomes in vaccinated thyroid cancer patients. Finally, we call for a prospective, multicenter, large-sample study to reveal the safety and immunogenicity of SARS-CoV-2 vaccines in thyroid cancer patients, especially for the different types of SARS-CoV-2 vaccines.

## Conclusions

In conclusion, inactivated whole-virion SARS-CoV-2 vaccines (CoronaVac and BBIBP-CorV) and recombinant protein vaccines (ZF2001) are safe in thyroid cancer patients. The regression time of SARS-CoV-2 IgG is significantly shorter in thyroid cancer patients than in healthy adults. Therefore, a booster vaccination dose may be earlier than the routine strategy for thyroid cancer patients.

## Data Availability Statement

The raw data supporting the conclusions of this article will be made available by the authors, without undue reservation.

## Ethics Statement

The studies involving human participants were reviewed and approved by the Second Affiliated Hospital of Chongqing Medical University. The patients/participants provided their written informed consent to participate in this study.

## Author Contributions

Conception and design: YH, JY, SF, GY, and DH. Administrative support: GY and DH. Provision of study materials or patients: DH, YH, JY, YF, and YM. Collection and assembly of data: DH, SF, JY, and YM. Data analysis and interpretation: YM, XL, YH, and JY. All authors contributed to the article and approved the submitted version.

## Funding

This work was supported by the National Natural Science Foundation of China (NSFC No. 81972460) for GY.

## Conflict of Interest

The authors declare that the research was conducted in the absence of any commercial or financial relationships that could be construed as a potential conflict of interest.

## Publisher’s Note

All claims expressed in this article are solely those of the authors and do not necessarily represent those of their affiliated organizations, or those of the publisher, the editors and the reviewers. Any product that may be evaluated in this article, or claim that may be made by its manufacturer, is not guaranteed or endorsed by the publisher.
